# miR-1/AMPK-Mediated Glucose and Lipid Metabolism under Chronic Hypothermia in the Liver of Freshwater Drum, *Aplodinotus grunniens*

**DOI:** 10.3390/metabo12080697

**Published:** 2022-07-27

**Authors:** Jianxiang Chen, Changyou Song, Haibo Wen, Guangxiang Liu, Ningyuan Wu, Hongxia Li, Miaomiao Xue, Pao Xu

**Affiliations:** 1 Wuxi Fisheries College, Nanjing Agricultural University, Wuxi 214081, China; 2020113020@stu.njau.edu.cn (J.C.); songchangyou@ffrc.cn (C.S.); wenhb@ffrc.cn (H.W.); 2019113019@stu.njau.edu.cn (G.L.); 2020813059@stu.njau.edu.cn (N.W.); 2021113016@stu.njau.edu.cn (M.X.); 2 Key Laboratory of Freshwater Fisheries and Germplasm Resources Utilization, Ministry of Agriculture and Rural Affairs, Freshwater Fisheries Research Center, Chinese Academy of Fishery Sciences, Wuxi 214081, China

**Keywords:** hypothermia, glucose and lipid metabolism, miR-1, AMPK, freshwater drum

## Abstract

Our previous study demonstrated that low temperature could induce hepatic inflammation and suppress the immune and oxidation resistance of freshwater drum. However, the metabolism, especially the glucose and lipid metabolism involved, is poorly studied. To further explore the chronic hypothermia response of freshwater drum, an 8-day hypothermia experiment was conducted at 10 °C to investigate the effect of chronic hypothermia on glucose and lipid metabolism via biochemical and physiological indexes, and metabolic enzyme activities, miRNAs and mRNA-miRNA integrate analysis in the liver. Plasma and hepatic biochemical parameters reveal chronic hypothermia-promoted energy expenditure. Metabolic enzyme levels uncover that glycolysis was enhanced but lipid metabolism was suppressed. Differentially expressed miRNAs induced by hypothermia were mainly involved in glucose and lipid metabolism, programmed cell death, disease, and cancerization. Specifically, KEGG enrichment indicates that AMPK signaling was dysregulated. mRNA-miRNA integrated analysis manifests miR-1 and AMPK, which were actively co-related in the regulatory network. Furthermore, transcriptional expression of key genes demonstrates hypothermia-activated AMPK signaling by miR-1 and subsequently inhibited the downstream glucogenic and glycogenic gene expression and gene expression of fatty acid synthesis. However, glycogenesis was alleviated to the control level while fatty acid synthesis was still suppressed at 8 d. Meanwhile, the gene expressions of glycolysis and fatty acid oxidation were augmented under hypothermia. In conclusion, these results suggest that miR-1/AMPK is an important target for chronic hypothermia control. It provides a theoretical basis for hypothermia resistance on freshwater drum.

## 1. Introduction

Water temperature is one of the most essential external factors for aquatic poikilotherms [1]. Temperature shifts outside of the optimal temperature range have negative impacts on the physiology and metabolism of the animal [2]. As a result, chronic exposure to suboptimal temperatures can also compromise the overall health of the animal [3]. Seasonal water temperature variances are major contributors to suboptimal temperatures. In China, water temperatures within a whole year vary enormously as the seasons change. In Wuxi, for example, the water temperature can reach 30 °C in summer while close to 0 °C in winter. Additionally, changes in latitude, topography, and precipitation contribute to the large temperature difference between the north and south of China. These have led to tremendous economic loss in the aquaculture industry every year. Therefore, the prevention and control of low temperatures are crucial to farming. In aquaculture, physical methods, crossbreeding, molecular markers, and nutritional enhancement are commonly used to improve fish resistance to low temperatures.

Low temperature stress induces a decrease in enzyme activity and cell membrane fluidity, resulting in cell functional damages, and ultimately impacts fish health and even individual death [4]. When cells suffer cold stimulation, the signal can be transmitted to nuclei via various stress pathways to initiate the stress response and establish new homeostasis [5]. To cope with hypothermia, energy expenditures are inevitable. Therefore, it is necessary to mobilize more endogenous energy materials to augment energy generation. There are a variety of studies suggesting that carbohydrate and lipid metabolism play important roles in the resistance to hypothermia. Glucose is generally considered the primary energy substance. Under low temperature stress, raising plasma glucose and enhanced glycolysis supply energy for life activities [6]. However, with the prolonging of time, plasma glucose content gradually drops below the normal level [7]. Meanwhile, lipid metabolism in fish is sensitive to temperature changes [8]. Cold stress promotes the synthesis of unsaturated fatty acids to strengthen fish adaptability to low temperatures [9].

AMPK, AMP-activated protein kinase, is common in eukaryotic organisms. AMPK is widely regarded as a switch that regulates cellular energy metabolism. AMPK can perceive variations in cellular energy metabolism and maintain the balance between energy supply and demand by multiple processes in cellular metabolism [10]. When intracellular adenosine triphosphate (ATP) is reduced, AMPK prompts glucose uptake and inhibits glycogen synthesis to promote glucose conversion to glycolysis [11,12]. Additionally, it has been suggested that AMPK inhibits acetyl-CoA carboxylase (ACC) activity through phosphorylation [13], which reduces malonyl-CoA synthesis to weaken the inhibition of carnitine o-palmitoyltransferase 1 (CPT1) [14,15], thereby enhancing fatty acid oxidation to generate more ATP. Furthermore, ACC is the rate-limiting enzyme in fatty acid synthesis, so AMPK is also involved in suppressing fatty acid synthesis [16].

More recently, omics approaches have become a research focus aiming at understanding the functions, mechanisms, and interactions as well as relationships between genes, transcripts, proteins, lipids, and other biomolecules [17]. Transcriptomics refers to a discipline on gene transcription and regulation in cells, which analyzes gene expression on the RNA level. RNA can be divided into messenger RNA (mRNA) and non-coding RNA (ncRNA) according to their structure and function. Among several types of ncRNAs, microRNAs (miRNAs) are a group of small RNAs (approximately 22-nt-long), which regulate gene expression mainly at the post-transcriptional level through translational repression or mRNA degradation [18]. Recent studies have highlighted that miRNAs play important roles in different stress adaptations [19,20]. Nevertheless, few authors reported on how low temperature stress affects fish miRNAs. It is, therefore, necessary to investigate the miRNAs and their target genes under low temperature stress.

Freshwater drum (*Aplodinotus grunniens*) is a kind of fish that is endemic to North and Central America. It is the only species in the genus of *Aplodinotus* that perpetually inhabits freshwater [21]. With respect to edibility, the freshwater drum is featured to possess a higher edible proportion, with delicious and nutritious flesh rich in proteins, amino acids, and fatty acids. Moreover, the freshwater drum has no intermuscular bones, which improves the fish quality and processing of the aquatic product [22]. With these prospects, we imported the larvae from the USA in 2016 and have achieved a milestone in artificial breeding and cultivation in 2019, providing a breakthrough and prospect for aquaculture. However, the mechanisms of hypothermia response in freshwater drum are still poorly understood. Summer water temperature in central and eastern China ranges from 25 to 35 °C, while the water temperature in the winter is generally below 10 °C. According to the previous study, the freshwater drum could adapt to the water temperature ranging from 7 to 30 °C [23]. Meanwhile, juvenile freshwater drum suffered increased mortality as the water temperature dropped to 1 °C and below [24]. Combined with our pre-experiment, as lukewarm water fish, the optimum temperature of freshwater drum should range from 18–26 °C. Therefore, it is necessary to study the low temperature response mechanism of freshwater drum for the development of aquaculture. This study from the perspective of metabolism was conducted to evaluate the effect of chronic hypothermia on glucose and lipid metabolism in livers of freshwater drum. In the present study, miRNA sequencing was applied to identify the key regulators under chronic hypothermia exposure. The epigenetic regulation between miRNA and encoding genes was also investigated. These results will suggest that glucolipid metabolism plays an important role in the low temperature response of freshwater drum. Meanwhile, these will provide a theoretical foundation for the regulation of miRNAs on glucose and lipid metabolism in freshwater drum under hypothermia exposure, which may provide a new method for the prevention and control of low temperature.

## 2. Results

### 2.1. Effects of Chronic Hypothermia on Plasma Biochemical Parameters of Freshwater Drum

**Figure 1:**
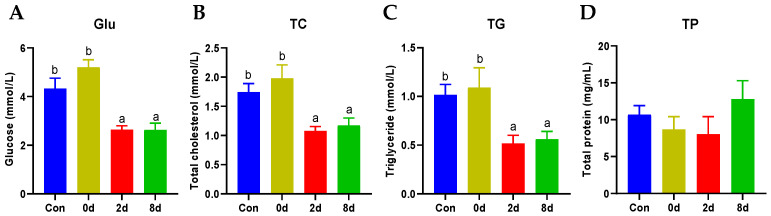
Effects of chronic hypothermia on plasma biochemical parameters of freshwater drum. (**A**) Glucose, Glu; (**B**) Total cholesterol, TC; (**C**) Triglyceride, TG; (**D**) Total protein, TP. Data were calculated by one-way ANOVA analysis with SPSS 23.0 (IBM SPSS Statistics, Version 23.0, Armonk, NY, USA). The significant differences are considered to exist when *p* < 0.05, and marked with different letters. Results were expressed as mean ± SEM, *n* = 6.

To explore the effects of chronic hypothermia on freshwater drum, plasma biochemical parameters were tested (shown in Figure 1). The levels of glucose (Glu), total cholesterol (TC), and triglyceride (TG) are elevated at the beginning of hypothermia. However, these significantly decreased at 2 d and 8 d (Figure 1A–C, *p* < 0.05). Unexpectedly, TP exhibited no statistical differences among all groups, though there was a downward trend at 2 d (Figure 1D, *p* > 0.05).

### 2.2. Effects of Chronic Hypothermia on Hepatic Biochemical Parameters of Freshwater Drum

**Figure 2:**
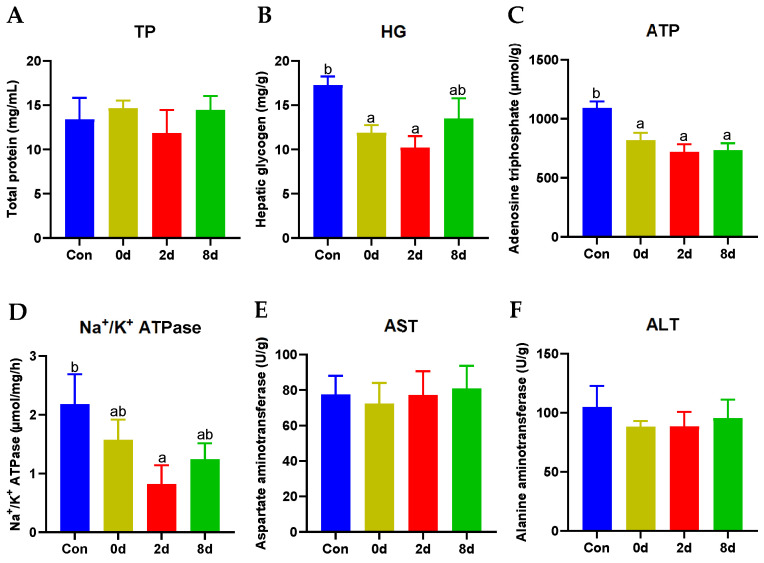
Effects of chronic hypothermia on hepatic biochemical parameters of freshwater drum. (**A**) Total protein, TP; (**B**) Hepatic glycogen, HG; (**C**) Adenosine triphosphate, ATP; (**D**) Na^+^/K^+^ ATPase. (**E**) Aspartate aminotransferase, AST; (**F**) Alanine aminotransferase, ALT. Data were calculated by one-way ANOVA analysis with SPSS 23.0. The significant differences are considered to exist when *p* < 0.05, and marked with different letters. Results were expressed as mean ± SEM, *n* = 6.

Hepatic biochemical parameters were next evaluated and shown in Figure 2. Hypothermia had no impact on TP level (Figure 2A, *p* > 0.05), which was in line with the result in Figure 1D. The levels of hepatic glycogen (HG) and Na^+^/K^+^ ATPase declined rapidly and reached a minimum at 2 d (Figure 2B,D, *p* < 0.05), while it approached to control level at 8 d (*p* > 0.05). Meanwhile, the regulation of adenosine triphosphate (ATP) was significantly inhibited in both groups (Figure 2C, *p* < 0.05). However, the activities of aspartate aminotransferase (AST) and alanine aminotransferase (ALT) were not impacted by hypothermia (Figure 2E,F, *p* > 0.05).

### 2.3. Effects of Chronic Hypothermia on Glucometabolism of Freshwater Drum

**Figure 3:**
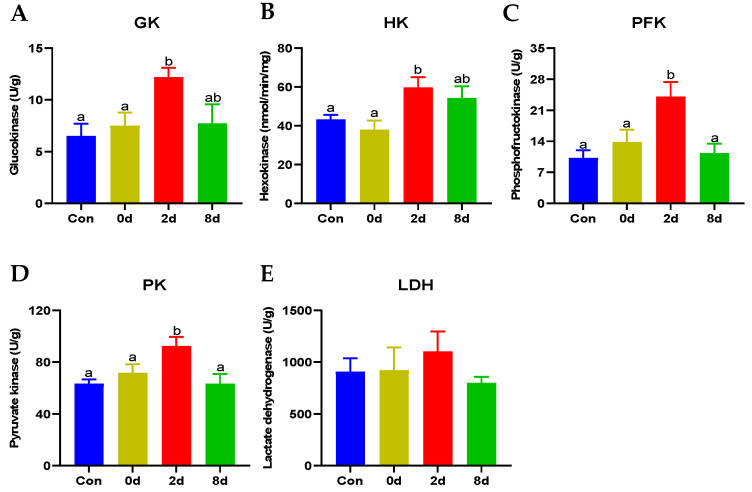
Effects of chronic hypothermia on glucometabolism of freshwater drum. (**A**) Glucokinase, GK; (**B**) Hexokinase, HK; (**C**) Phosphofructokinase, PFK; (**D**) Pyruvate kinase, PK; (**E**) Lactate dehydrogenase, LDH. Data were calculated by one-way ANOVA analysis with SPSS 23.0. The significant differences are considered to exist when *p* < 0.05, and marked with different letters. Results were expressed as mean ± SEM, *n* = 6.

To further explore the effects of hypothermia, enzymes on glucometabolism were tested (shown in Figure 3). The activities of enzymes on glycolysis including glucokinase (GK), hexokinase (HK), phosphofructokinase (PFK), and pyruvate (PK) rose remarkably and peaked at 2 d (Figure 3A–D, *p* < 0.05); thereafter, these enzymes decreased to levels that did not differ significantly in comparison to the control group with continued hypothermia. Meanwhile, the activity of lactate dehydrogenase (LDH), a key enzyme in anaerobic glycolysis, displayed no significant difference under low temperature (Figure 3E, *p* > 0.05).

### 2.4. Effects of Chronic Hypothermia on Lipid Metabolism of Freshwater Drum

**Figure 4:**
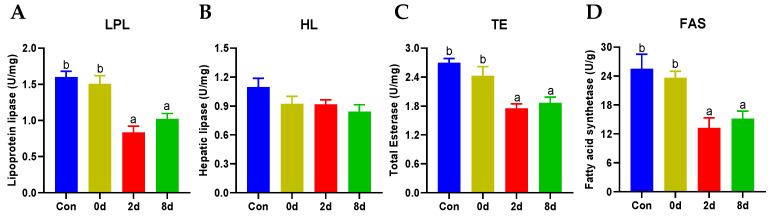
Effects of chronic hypothermia on lipid metabolism of freshwater drum. (**A**) Lipoprotein lipase, LPL; (**B**) Hepatic lipase, HL; (**C**) Total Esterase, TE; (**D**) Fatty acid synthetase, FAS. Data were calculated by one-way ANOVA analysis with SPSS 23.0. The significant differences are considered to exist when *p* < 0.05, and marked with different letters. Results were expressed as mean ± SEM, *n* = 6.

Enzymes on lipid metabolism were next evaluated (shown in Figure 4). The activities of lipoprotein lipase (LPL), total esterase (TE), and fatty acid synthetase (FAS) were inhibited significantly with prolonged hypothermia exposure (Figure 4A,C,D, *p* < 0.05). However, hepatic lipase (HL) displayed no significant differences in contrast to the control group (Figure 4B, *p* > 0.05).

### 2.5. miRNA Analysis Reveals AMPK Signaling Was Active to Glucose and Lipid Metabolism under Hypothermia Exposure

**Figure 5:**
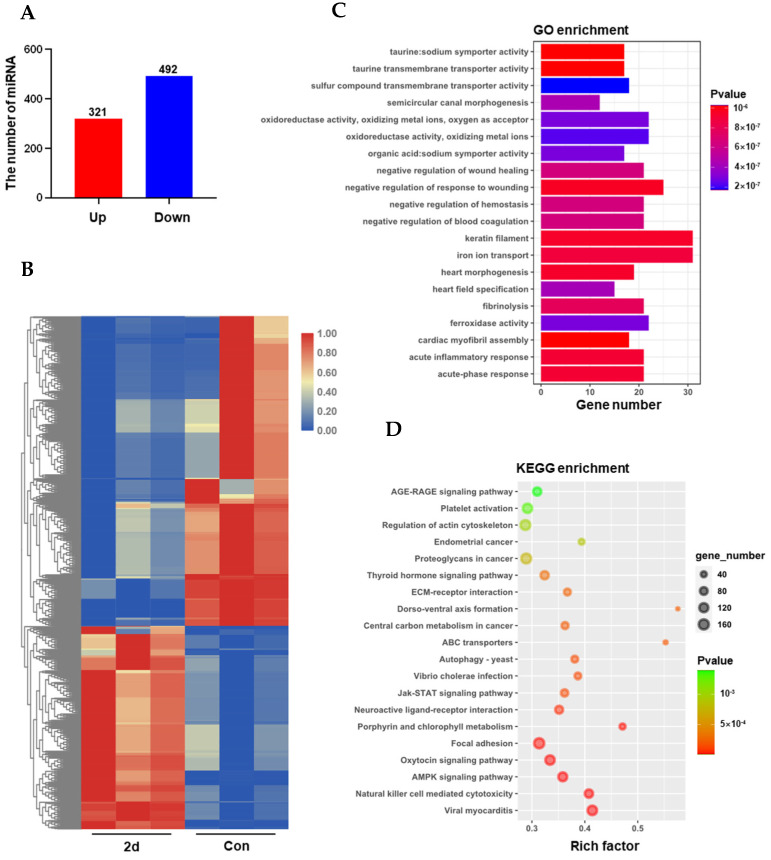
miRNA analysis reveals that AMPK signaling was active in glucose and lipid metabolism under hypothermia exposure. (**A**) up-and down-regulated differentially expressed miRNAs; (**B**) Hierarchical clustering of differentially expressed miRNAs among the 6 libraries; (**C**) GO enrichment of differentially expressed miRNAs 2 d vs. Con; (**D**) KEGG enrichment of differentially expressed miRNAs 2 d vs. Con.

To reveal the underlying mechanism of glucose and lipid metabolism under hypothermia, high-throughput sequencing was conducted to reveal the dynamically regulated miRNAs. According to the above results, the treatment group of 2 d was selected for research. We first used univariate statistical analyses, including fold change (FC) analysis and Students’ *t*-test. In comparison with the control group, a total of 813 differentially expressed miRNAs (DEMs) at 2 d was identified with the threshold of |log_2_FC| > 1 and *p* < 0.05, including 321 up-regulated and 492 down-regulated DEMs (Figure 5A). Meanwhile, these 813 DEMs were clustered into different subclusters according to their expression levels in the heatmap (Figure 5B).

To uncover the underlying regulation of these miRNAs, target prediction was conducted with the background of RNA-seq results. The obtained target differentially expressed RNAs (DEGs) were subjected to the Gene Ontology (GO) and Kyoto Encyclopedia of Genes and Genomes (KEGG) analysis. GO enrichment indicated the target genes were mainly involved in the biological process of acute-phase response (GO:0006953), acute inflammatory response (GO:0002526), iron ion transport (GO:0006826), cellular component of keratin filament (GO:0045095), molecular function of oxidoreductase activity (GO:0016722), ferroxidase activity (GO:0004322), and ATP binding (GO:0005524) (Figure 5C, Supplementary Material Table S1). Meanwhile, KEGG enrichment demonstrated that these target DEGs were enriched in carbohydrate and lipid metabolism (AMPK signaling pathway, Insulin signaling pathway, Glycolysis/Gluconeogenesis, Fat digestion and absorption, Glycerophospholipid metabolism), programmed cell death (Ferroptosis and Apoptosis), disease (Vibrio cholerae infection, Type II diabetes mellitus, Epstein-Barr virus infection and Graft-versus-host disease), and cancerization (Proteoglycans in cancer, Endometrial cancer, and Non-small cell lung cancer) (Figure 5D, Table S2).

### 2.6. mRNA-miRNA Integrate Analysis Reveals miR-1 and AMPK Were Involved in Hypothermia Exposure

**Figure 6:**
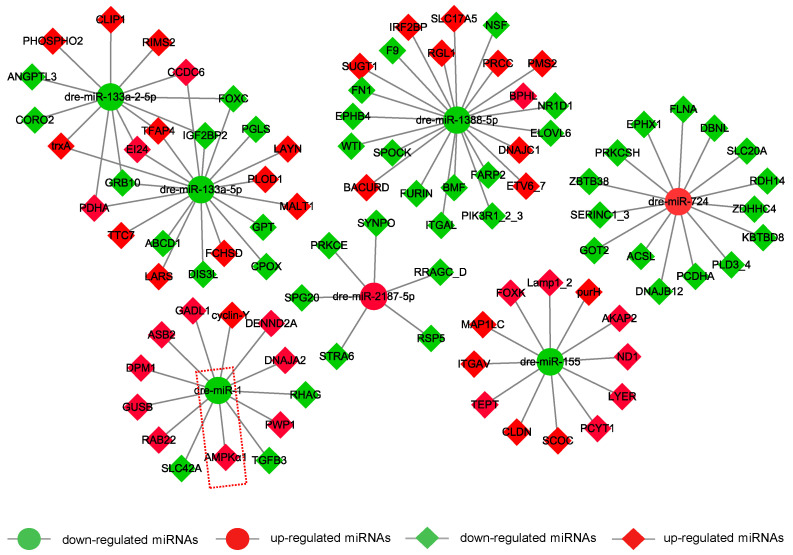
mRNA-miRNA integrate analysis reveals miR-1 and AMPK were involved in hypothermia exposure. Sig-diff miRNAs were selected to conduct mRNA-miRNA integrate analysis, and DEGs were targeted as the background. Data retrieved from high-throughput sequencing were applied to the integrated network by Cytoscape 3.7.2. The red dotted box represents there was a targeting relationship between miR-1 and AMPKα1.

To further explore the potential regulatory mechanism, mRNA-miRNA-integrated network analysis was performed. As shown in Figure 6, seven DEMs play important roles in the network with their target genes. Concomitantly, miR-1, miR-133a-5p, miR-133a-2-5p, miR-1388-5p, miR-2187-5p, miR-724, and miR-155 were mutually regulated by targeting to specific encoding genes. In particular, we found that miR-1/AMPK was involved in hypothermia response.

### 2.7. AMPK Signaling Was Dysregulated under Hypothermia Based on Transcriptomic Analysis

**Figure 7:**
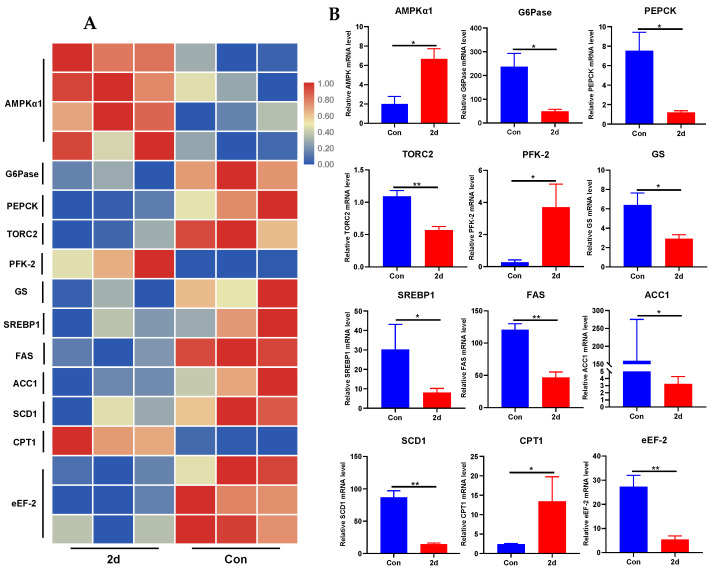
AMPK signaling was dysregulated under hypothermia based on transcriptomic analysis. (**A**) heatmap; (**B**) transcriptional expression of key genes in AMPK signaling pathway retrieved from the transcriptome. Data were analyzed by Student’s *t*-test with SPSS 23.0, asterisk represents the statistical difference (*, *p* < 0.05; **, *p* < 0.01). Results were expressed as mean ± SEM, *n* = 3.

To uncover whether AMPK signaling was active in hypothermia, we next quantified the relative expression of the key genes in AMPK signaling from the RNA-seq databases that were constructed previously. From the heatmap cluster (Figure 7A) and relative expression levels (Figure 7B), AMPK, PFK-2 and CPT1 were activated (*p* < 0.05), while G6Pase, PEPCK, TORC2, GS, SREBP1, FAS, ACC1, SCD1 and eEF-2 were significantly inhibited (*p* < 0.05).

### 2.8. miR-1/AMPK Signaling Was Involved in Glucose and Fatty Acid Metabolism under Chronic Hypothermia

**Figure 8:**
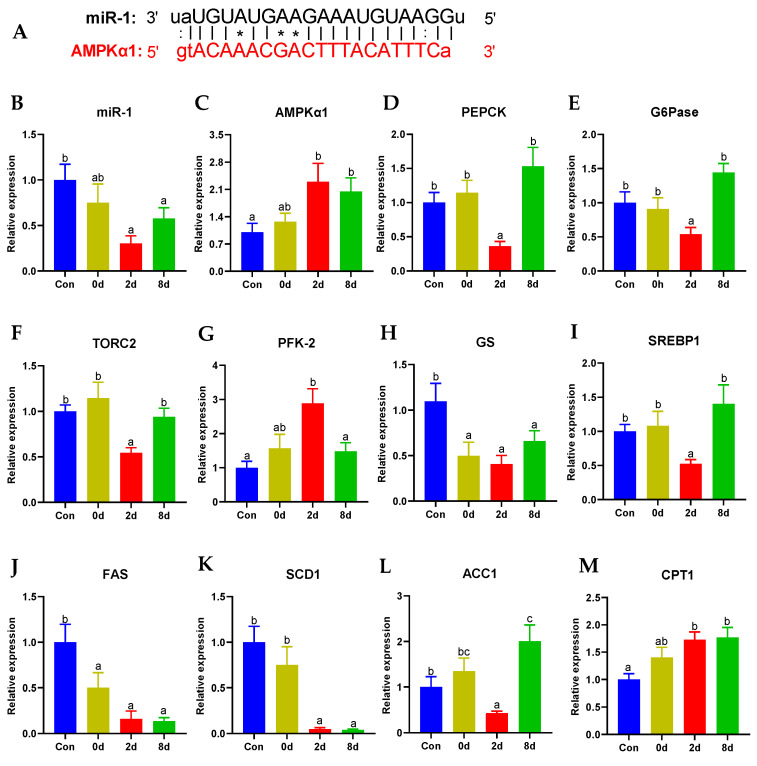
miR-1/AMPK signaling was involved in glucose and fatty acid metabolism under chronic hypothermia. (**A**) target prediction between miR-1 and AMPKα1; (**B**) miR-1; (**C**) AMP-activated protein kinase, AMPK; (**D**) Phosphoenolpyruvate carboxykinase, PEPCK; (**E**) Glucose-6-phosphatase, G6Pase; (**F**) CREB-regulated transcription coactivator 2, TORC2; (**G**) 6-phosphofructo-2-kinase-2, PFK-2; (**H**) Glycogen synthase, GS; (**I**) Sterol regulatory element-binding transcription factor 1, SREBP1; (**J**) Fatty acid synthase, FAS; (**K**) Stearoyl-CoA desaturase 1, SCD1; (**L**) Acetyl-CoA carboxylase 1, ACC1; (**M**) Carnitine O-palmitoyltransferase 1, CPT1. Data were calculated by one-way ANOVA analysis with SPSS 23.0. The significant differences are considered to exist when *p* < 0.05, and marked with different letters. Results were expressed as mean ± SEM, *n* = 9.

Target predictions indicate that AMPKα1 was the target gene of miR-1 (Figure 8A). To further evaluate whether the AMPK signaling pathway was involved in glucose and lipid metabolism under chronic hypothermia, we investigated the transcriptional expression of key genes. From the results, the regulation between miR-1 and AMPK under chronic hypothermia was confirmed, indicating that the expression of miR-1 was inhibited by hypothermia and persisted up to 8 days (*p* < 0.05, Figure 8A). Synchronously, the expression of AMPK was also activated at 2 d and 8 d *(p* < 0.05, Figure 8B).

Apart from miR-1 and AMPK, gene expressions of PEPCK, G6Pase, and TORC2 on gluconeogenesis and GS on glycogen synthesis were significantly inhibited at 2 d (Figure 8C–E,G, *p* < 0.05), while the expression of PFK-2 on glycolysis was activated (Figure 8F, *p* < 0.05). Importantly, the expression of these genes at 8 d showed no significant changes apart from GS (Figure 8C–G, *p* > 0.05). Additionally, the mRNA expressions of SREBP1 and ACC1 demonstrated remarkable decreases at 2 d (Figure 8H,K, *p* < 0.05), while it notably increased after hypothermia for 8 days (Figure 8H,K, *p* < 0.01). Simultaneously, the gene expressions of FAS and SCD1 declined significantly throughout the chronic hypothermia (Figure 8I,J, *p* < 0.05). However, the mRNA expression of CPT1, a crucial gene for fatty acid oxidation, promptly elevated in both groups (Figure 8L, *p* < 0.05).

### 2.9. Epigenetic Schematic of AMPK Signaling under Hypothermia Induction in Freshwater Drum

**Figure 9:**
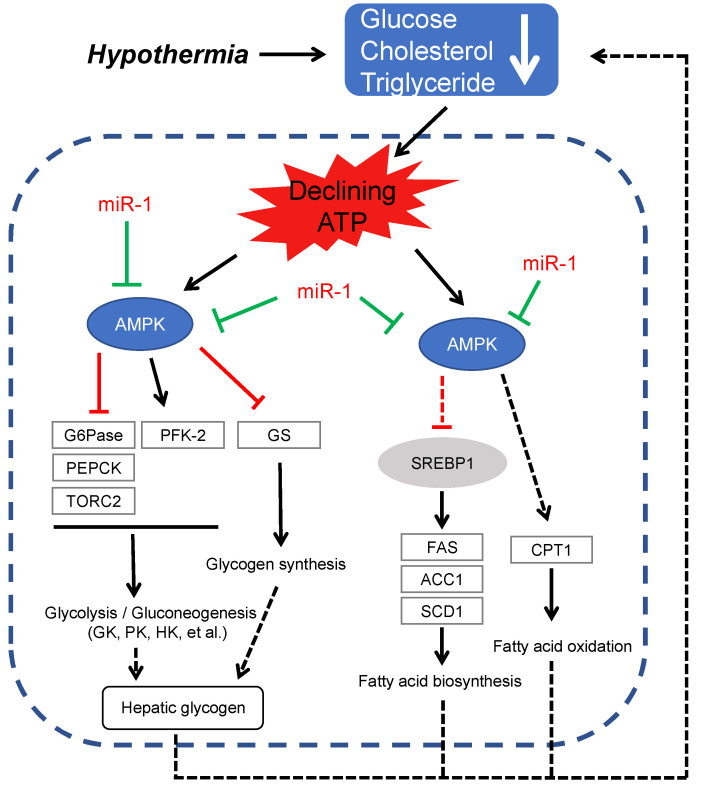
Epigenetic schematic of AMPK signaling under hypothermia induction in freshwater drum. miR-1 negatively regulates AMPK. This epigenetic network proves miR-1/AMPK is the important target for glucose and lipid metabolism under hypothermia response for freshwater drum.

In the present study, we validated the relationship between miRNAs and target genes with high throughput sequencing, target prediction, and RT-PCR. Based on the above results, we raise the hypothetical regulation schematic of miR-1/AMPK signaling on glucose and lipid metabolism under hypothermia (Figure 9). Hypothermia inhibited miR-1 and subsequently activated AMPK signaling and downstream modulators to affect glucose and lipid metabolism on freshwater drum.

## 3. Discussion

Freezing in winter takes a heavy toll on the Chinese aquaculture industry every year [25]. This study was conducted to investigate the effects of chronic low temperature on carbohydrate and lipid metabolism in freshwater drum. Some studies have demonstrated that water temperature is an important factor affecting fish physiology [26]. In the present study, the content of ATP and Na^+^/K^+^ ATPase in the liver decreased remarkably under chronic hypothermia exposure. The freshwater drum resists chronic hypothermia by a large amount of energy expenditure to maintain normal physiological activities.

Glucose is one of the main energy sources. Glucometabolism is divided into catabolism and anabolism. Glycolysis and gluconeogenesis are two opposite metabolic pathways, which could be mutually regulated, and so the activation or inhibition of key enzymes in the two metabolic pathways can cooperate [27,28]. In the present study, glucose and hepatic glycogen levels further corroborated that freshwater drum expended lots of energy at chronic low temperatures. Glucometabolism was involved in hypothermia exposure but slowly returned to a normal level over time. Glucose can provide energy for stress response in freshwater drum at the early stage of hypothermia exposure. Chronic hypothermia-promoted glycolysis thereinto aerobic glycolysis was predominant. Inversely, gluconeogenesis was suppressed. These reactions are the self-protection for freshwater drum under stress environment by increasing energy production and reducing energy consumption to adapt to chronic hypothermia [29].

Lipids are important energy storage substances in animals [30]. Lipids play an important role in fish health; lipid metabolic disorders can directly affect fish growth, development, and physiological activities, including its stress resistance [31]. Among them, triglyceride is an important form of energy storage, and it is oxidized for energy supply. Triglycerides are hydrolyzed to fatty acids and glycerol by lipase such as LPL [32], and then fatty acids are β-oxidized to acetyl-CoA, using the energy supply in the tricarboxylic acid cycle [33]. Simultaneously, acetyl-CoA is also the raw material for fatty acids and cholesterol synthesis [34,35]. Under the catalysis of FAS and other synthases, plenty of ATPs are consumed to synthesize fatty acids in the liver [35]. In this present study, chronic hypothermia decreased plasma triglyceride and cholesterol levels. The freshwater drum was kept in a state of energy deficiency under chronic hypothermia. Glucometabolism failed to meet enough energy supply under stress. Therefore, the decomposition of triglycerides and other lipids was supposed to participate in the regulation of energy homeostasis [36]. Meanwhile, cholesterol is a sterol type of lipid that serves as an essential structural component of animal cell membranes. Chronic hypothermia exposure inevitably suppressed cholesterol synthesis and could even deplete cholesterol to disrupt cell membrane function. Lipolysis-related enzymes and fatty acid synthase in the liver were inhibited, and these were not alleviated over time. Hypothermia caused effects on the rapid reduction of perivisceral lipids and non-polar lipid deposition in liver [37]. As a result, the strong reduction, particularly in LPL activity, may correspond to a form of liver protection, limiting lipid uptake from plasma lipoproteins [38]. Synchronously, chronic hypothermia tends to restrain the activity of fatty acid synthase to maintain energy balance. Therefore, lipid metabolism persisting up to the end of the experiment may be more necessary than glucometabolism at chronic low temperatures.

Protein is an important nutrient in the organism. All the structures and metabolism in an organism require the participation of protein. Under stress, aquatic animals can provide energy by proteolysis in different organs [39,40]. AST and ALT are the most important aminotransferases in the liver, which are related to protein catabolism [41,42]. In this experiment, TP in plasma and liver both remained unchanged. Similarly, there were no changes in ALT and AST. These results indicate that chronic hypothermia did not cause functional damage to the liver in freshwater drum. Proteolysis is not the main way to supply energy to maintain physiological activity in freshwater drum during chronic low temperature exposure.

Determining the miRNA profiles under stress is extremely valuable to the characterization of the regulation of biological functions [43]. Previous studies have demonstrated that miRNAs are closely related to temperature stress [44,45]. To further reveal the underlying epigenetic mechanism, the integrated analysis between miRNAs and target mRNAs was conducted with throughput sequencing in the present study. The results reveal that AMPK signaling was activated to cope with chronic hypothermia stress. Specifically, miR-1 targeted AMPK signaling was involved in the regulatory network. Transcriptomic analysis reveals that the downstream target genes were dysregulated under hypothermia stress. These data confirm the prospect that AMPK is the target in response to hypothermia.

It is reported that miR-1 is closely related to glucose and lipid metabolism. Sun et al. [46] identified that there was a significant decrease in the levels of miR-1 involved in glycolysis under temperature stress. In the present study, miR-1 was inhibited under hypothermia, and the mRNA-miRNA integrate analysis reveals that miR-1 and AMPK were involved in chronic hypothermia stress, and the contrasting expression of miR-1 and AMPK indicate that miR-1 negatively regulates AMPK under hypothermia. AMPK is a crucial molecule in the regulation of biological energy metabolism. Jia et al. [47] reported a miR-1-mediated AMPK pathway to inhibit rat cardiac fibroblasts fibrosis induced by high glucose. However, in another study, HIF-1α was demonstrated to be a direct functional target of miR-1. The downregulation of miR-1 significantly increased HIF-1α expression, resulting in enhanced tumor glycolysis [48]. Taken together, these findings suggest that miR-1 is a critical regulator of fundamental biological processes in freshwater drum under chronic hypothermia.

To further discuss whether miR-1/AMPK medicated the carbohydrate and lipid metabolism under chronic hypothermia stress, the transcriptional expression of downstream genes in the AMPK signaling pathway was evaluated with RT-PCR. The results indicate that the AMPK signaling pathway was activated. Hypothermia promoted the regulation of glycolysis, and fatty acid oxidation while inhibiting glycogen synthesis, gluconeogenesis, and fatty acid synthesis. Over time, glucometabolism was alleviated, while lipid metabolism was still affected. Notably, the gene expressions of SERBP1 and ACC1 were up-regulated after hypothermia for 8 days, meaning chronic hypothermia potentially promoted the synthesis of unsaturated fatty acids to strengthen fish adaptability. Collectively, these results further demonstrate that chronic hypothermia impacts the regulation of carbohydrate and lipid metabolism depending on the AMPK mediated by miR-1. Moreover, the lipid metabolism has a significant implication in the chronic hypothermia response of freshwater drum, and even lipid consumption can last through the whole overwintering period [49], which is consistent with the results of metabolic enzyme activities. Together, these results will provide a reference for the prevention and control of low temperature on freshwater drum.

## 4. Materials and Methods

### 4.1. Ethics Statement

This study was approved by the Animal Care and Use Committee of Nanjing Agricultural University (Nanjing, China). All animal procedures were performed according to the Guideline for the Care and Use of Laboratory Animals in China.

### 4.2. Experimental Animals and Rearing Conditions

The hypothermia experiment was conducted at Wuxi Fisheries College of Nanjing Agricultural University. Laboratory fish were the first-generation larvae of freshwater drum introduced from the United States by the Freshwater Fisheries Research Center, Chinese Academy of Fishery Sciences. Freshwater drum were reared in a temperature-adjustable circulating water system (specifications for φ 820 × 700 mm) consisting of 12 tanks (300 L each). Prior to the experiment, fish were acclimated in the tank fed with fresh bait at 25 °C for 14 days. After acclimation, fish averaging 20.88 ± 2.75 g were randomly assigned into 9 tanks (3 tanks per group, 20 fish per tank) and were fed with fresh bait (3–5% of their body weight) twice a day (8:00 and 16:00). During the 8-day experiment, we cleaned up food scraps and feces daily. During the experiment, the temperature gradually decreased from 25 °C to 10 °C in 15 h at a rate of 1 °C/h and was then maintained at 10 °C for 0 d, 2 d and 8 d. A temperature of 25 °C was set as the control group (Con). Throughout the experiment, dissolved oxygen was kept as >6 mg L^−1^, pH 7.2–7.8, NO_2_^−^ < 0.02 mg L^−1^, and NH_3_ < 0.05 mg L^−1^.

### 4.3. Sample Collection

Experimental samples were collected at 0 d, 2 d and 8 d. Fish were starved for 24 h to evacuate the alimentary tract contents before sampling. Fifteen fish from each tank were randomly sampled and anesthetized with MS-222 (100 mg L^−1^) at each time point. Blood samples were obtained from the caudal vein and injected into anticoagulant tubes. These samples were centrifuged at 5000 rpm at 4 °C for 10 min to extract the plasma. The plasma was stored at −80 °C for the measurement of biochemical indicators. Meanwhile, the sampled fish were dissected to collect the liver on ice, froze in liquid nitrogen immediately, and stored at −80 °C for subsequent analysis.

### 4.4. Plasma and Liver Biochemical Indicators Analysis

Plasma samples of two fish from each tank were used to measure parameters including glucose (Glu), total cholesterol (TC), triglyceride (TG), and total protein (TP). Additionally, liver samples of two fish from each tank were used to measure parameters including total protein (TP), hepatic glycogen (HG), adenosine triphosphate (ATP), Na^+^/K^+^ ATPase, aspartate aminotransferase (AST), alanine aminotransferase (ALT), glucokinase (GK), hexokinase (HK), phosphofructokinase (PFK), pyruvate kinase (PK), lactate dehydrogenase (LDH), lipoprotein lipase (LPL), hepatic lipase (HL), total esterase (TE) and fatty acid synthetase (FAS) according to the manufacturer’s instructions. All the assay kits were purchased from Nanjing Jiancheng Bioengineering Institute, China. In detail, Glu was detected by the hexokinase method (Category No: F006-1-1), TC was determined by the colorimetric method (Category No: A111-2), TG was determined by the colorimetric method (Category No: A110-2), TP was determined by Coomassie brilliant blue (Category No: A045-2), HG was determined by colorimetric method (Category No: A043-1-1), ATP was determined by colorimetric method (Category No: A095-1-1), Na^+^/K^+^ ATPase was determined by colorimetric method (Category No: A016-1), AST was determined by Reit′s method (Category No: C010-1-1), ALT was determined by Reit′s method (Category No: C009-1-1), GK was determined by enzyme linked immunosorbent assay (ELISA) (Category No: H439-1), HK was determined by UV-spectrophotometric method (Category No: A077-3-1), PFK was determined by ultraviolet colorimetry method (Category No: A129-1-1), PK was determined by ultraviolet colorimetry method (Category No: A076-1-1), LDH was determined by colorimetry method (Category No: A020-1-2), LPL, HL and TE were determined by colorimetry method (Category No: A067-1-2), and FAS was determined by ELISA (Category No: H231-1-1).

### 4.5. RNA Extraction and De Novo High-Throughput Sequencing

#### 4.5.1. RNA Extraction, cDNA Library Construction, and RNA-Seq

The total liver RNA of each group was extracted using TRIzol Reagent according to protocols (Takara, Dalian, China). In each group, nine liver tissues were selected to conduct the high-throughput sequence, wherein three fish in each group were randomly mixed and three biological replicates were finally applied for RNA-seq. After the qualified three micrograms of total RNA from each sample, ployA by magnetic beads with Oligo (dT) can be used to isolate mRNA from total RNA. The mRNA could be randomly fractured by fragmentation buffer, and small fragments of about 300 bp could be separated by magnetic bead screening. RNA fragments were converted to cDNA using random primers, followed by second-strand cDNA synthesis and end repair. End Repair Mix was added to fill the sticky end of the double-stranded cDNA into the flat end, and then base A was added to the 3′ end to connect the Y-shaped joint. Adaptor-tagged cDNA fragments were enriched using the manufacturer’s cocktail and 15-cycle PCR. The target bands were recovered from 2% agarose gel and PCR amplification to obtain the final sequencing libraries. Illumina Hiseq6000 was used for sequencing after the library was qualified by quality inspection. Sequencing reads are paired-end 2 × 150 bp (PE150).

#### 4.5.2. De Novo Assembly, Functional Annotation and the Differentially Expressed Genes (DEGs) Analysis

Unqualified raw data was filtered into clean data using cut adapt software before being assembled, including removing the joint sequences in reads and the reads without inserted fragments due to self-connection of the joint, removing low qualities reads (bases of Q ≤ 10 ranks over 20% of the whole read), removing reads with over 10% N, cutting the adapters and the less than 30 bp sequences. All sequences after quality control generated contig and singleton through de novo assembly, which were finally connected to obtain transcripts. All transcripts were compared with six databases (NR, Swiss-PROT, Pfam, COG, GO, and KEGG databases) to obtain functional annotation information. The transcriptome was quantified by RSEM. The DEGs were identified based on Fragments Per Kilobase of exon model per Million mapped reads (FPKM). As the sequencing depth of samples differs from each other, the absolute gene expression was normalized to the FPKM value, which made FKPM the expression quantity of genes. Deseq2 was used to analyze the variation on DEGs (the significant difference threshold was |log_2_FC| > 1, *p* < 0.05). GO term and KEGG pathway enrichment were conducted to analyze DEGs and pathways.

### 4.6. miRNA Sequencing, Identification, and Target Gene Prediction

#### 4.6.1. miRNA Sequencing

The Con and 2 d groups were applied for miRNA analysis. In each group, nine liver tissues were selected to conduct the high-throughput sequence, wherein three fish in each group were randomly mixed and three biological replicates were finally applied. After total RNA was extracted, 3′ and 5′ adaptors were added to synthesize the transcript into cDNA. After PCR amplification, fragments between 140 and 150 bp were selected for miRNA sequencing with Illumina Hiseq6000 platform. Fastx-Toolkit (Majorbio Bio-pharm Technology Co., Ltd, Shanghai, China) was applied to remove adapters, lower quality bases (mass value less than 20), and too long or too short reads (more than 32 nt and less than 18 nt). The clean readings were mapped to NR, Rfam, and miRbase for annotation (less than 2 mismatches).

#### 4.6.2. Differentially Expressed miRNAs (DEMs) Identification and Target Gene Prediction

Clean reads were compared with miRBase and Rfam databases to obtain known miRNA annotation information. New miRNAs were predicted from reads without annotated information by miRDeep2 software. Reads with |log_2_FC| > 1 and *p* < 0.05 were identified as DEMs. The database miRanda, targetscan, and RNAhybrid were applied for target gene prediction. Targeted genes with corrected *p* < 0.05 (corrected with Bonferroni) were identified as targeted DEGs, and target DEGs were also subjected to Gene Ontology (GO) and KEGG enrichment analysis with Blast2GO.

### 4.7. Validation of Differentially Expressed Genes Obtained from RNA-Seq

Real-time quantitative PCR (RT-PCR) was conducted according to our previously established methods [50] to validate the expressions of key genes involved in glucose and lipid metabolism (*n* = 9). All the primers were synthesized in Sangon Biotech Co., Ltd. (Shanghai, China). Details of primers are listed in Table 1. RT-PCR was performed with SYBR Green (Takara, Dalian, China) on Takara 800 Fast Real-Time PCR System according to the manufacturer’s protocol.

### 4.8. Statistical Analysis

All data in the study were represented as mean ± standard error mean (SEM), which was calculated using SPSS software (version 23.0). The data of plasma, liver biochemical indicators, and metabolic enzyme activity were analyzed with one-way analysis of variance (ANOVA) followed by a Duncan multiple-range test. Independent samples *t*-test was conducted to analyze the transcriptional expression of detected genes based on the transcriptome. Relative RNA expression was calculated using the 2^−ΔΔCT^ comparative CT method and analyzed with one-way analysis of variance (ANOVA) followed by a Duncan multiple-range test.

## 5. Conclusions

In this study, chronic hypothermia stress-induced alteration in glucose and lipid metabolism in freshwater drum. Lipid metabolism plays a critical role in the chronic hypothermia response of freshwater drum. miRNA sequencing and integrated analysis with mRNAs conjointly reveal that miR-1/AMPK was involved in hypothermia stress. Transcriptional expression uncovers the miR-1/AMPK contributions to the regulation of carbohydrate and lipid metabolism to maintain normal physiological activities in freshwater drum under chronic hypothermia stress. In conclusion, miR-1/AMPK could be an important target for chronic hypothermia responses in freshwater drum.

## Figures and Tables

**Table 1:** Primers and sequences referred in the experiments.

Gene	Primer	Sequence (5′-3′)
miRNA primer		
miR-1	F	CGGGAATGTAAAGAAGTATG
U6	F	CTCGCTTCGGCAGCACA
	R	AACGCTTCACGAATTTGCGT
mRNA primer		
AMPK	F	TCCCTCCTACAGCAACAAC
	R	GACGCCAGGTAGAAATCC
PEPCK	F	TCGTCTCATTCGGTAGTGGT
	R	CGTAGGTTGCCTTGGTTGT
G6Pase	F	GCGGACCTGAGGAACACCTT
	R	AAACACCTGCGGCTCCCATA
TORC2	F	AAGTTTAGCGAGAAGATAGCG
	R	TCAAGAGTGGATGGGAAGG
PFK-2	F	CCAGATAATGAGGAGGGTTT
	R	TCTTGTTGTATTTGTGGCATC
GS	F	GAGCCTCCGCACATCGTA
	R	CGCCTGCTTCCTTATCCA
SREBP1	F	TTCCTCTCCCTCAACCCTCT
	R	TTACGGGCTCTCCATACACC
FAS	F	TGGCATCGAGTACAACAAGC
	R	TTGGCACGAAGTAGCATCAC
SCD1	F	CGGGGCTTCTTCTTCTCTCA
	R	GAAGCACATGACTAGCACGG
ACC1	F	CTGGAGGAGACGGTGAAAAG
	R	TGCGTATCTGCTTGAGGATG
CPT1	F	GCACCAGAACCTTTACCGA
	R	TCCGCCCAGTGATATGAAC
eEF-2	F	TCAGCCCTGTGGTGAGAAT
	R	TGTGCTTGTTGGGTGACTTT
β-actin	F	AGGCTGTGCTGTCCCTGTAT
	R	GCTGTGGTGGTGAAGGAGTAG

Note: The mRNA sequences for each gene were obtained from freshwater drum liver transcriptome sequencing database of the Gene Center of Freshwater Fisheries Research Center, Chinese Academy of Fishery Sciences. Primers for RT-PCR were designed using Primer premier 5.0.

## Data Availability

The data that support the findings of this study are available from the corresponding author upon reasonable request.

## Supplementary Materials

The following supporting information can be downloaded at: https://www.mdpi.com/article/10.3390/metabo12080697/s1, Table S1: GO enrichment analysis of differentially targeted genes on DEMs; Table S2: KEGG enrichment analysis of differentially targeted genes on DEMs.
